# Clinical Application of Mesenchymal Stem Cells in the Treatment and Prevention of Graft-versus-Host Disease

**DOI:** 10.1155/2011/427863

**Published:** 2011-12-10

**Authors:** Yi Lin, William J. Hogan

**Affiliations:** Division of Hematology, Department of Medicine, Mayo Clinic, 200 1st Street SW, Rochester, MN 55905, USA

## Abstract

Mesenchymal stem cells (MSCs) represent a heterogeneous population of stromal cells with pluripotent mesenchymal differentiation potential. They have been found to have immunosuppressive properties and the ability to modulate angiogenesis and endogenous tissue repair by *in vitro* and animal studies. Clinical trials have examined the utility of these cells in autoimmune and inflammatory conditions. In particular, in allogeneic hematopoietic stem cell transplant (HSCT), multiple studies have been conducted to explore the use of MSC to treat acute and chronic graft-versus-host disease (GVHD) and for cotransplantation with HSCT to promote HSC engraftment and prevent GVHD. We review here the results of these studies and discuss some challenges of this treatment modality in this disease setting.

## 1. Introduction

Mesenchymal stem cell and multipotent mesenchymal stromal cells are both designated MSC nomenclature by the latest consensus statement from the International Society for Cellular Therapy (ISCT) [1]. This is a group of heterogeneous plastic-adherent cells that can be isolated from bone marrow (BM), adipose tissue, placenta, cord blood, and other tissues. The name MSC simply implies the mesenchymal origin of these cells and is not necessarily the limit of their differentiation potential. Given the heterogeneity of the stromal cell compartment and the limited number of these cells that have true stem-cell-like properties, consistent characterizations of MSC were proposed to maximize intersample equivalency in data comparison [2]. Three criteria are now commonly used among researchers:

1. adherence to plastic in *in vitro* culture,
2. surface antigen expression positivity (*⩾*95%) for CD 105, CD73, CD90 and negativity (*⩽*2%) for lineage markers including CD45, CD34, CD14 or 11b, CD79 alpha or CD19, and HLA-DR,
3. capacity for differentiation *in vitro* into osteoblasts, adipocytes, and chrondroblasts.

*In vitro* MSC have been shown to exert immunosuppressive effects via direct suppression of T and B lymphocytes, NK cell, and dendritic cell functions [3–12]. They can also secrete cytokines important for angiogenesis, tissue repair, and immune modulation such as VEGF, IL-6, IL11, M-CSF, and stem cell factor [13–15]. In animal models, some reports suggest prolonged survival of skin and solid organ grafts with MSC infusion [4, 16–18] while protection from graft-versus-host disease (GVHD) by MSC in the mouse model is less clear [19–21]. This is partially attributed to the complex biology of these cells compounded by variations in activation of their potential functions by different culture techniques across different labs. Interspecies differences in MSC functions and discrepancies between *in vitro* and *in vivo* studies have also been described [22]. 

In recent decade, there has been intense interest in clinical applications of MSC for modulation of immunity and endogenous repair. At the time of writing, there are currently 179 clinical trials using MSC registered at clinicaltrials.gov. Crohn's disease, cardiac ischemia, limb ischemia, amyotrophic lateral sclerosis, diabetes, multiple sclerosis, and liver cirrhosis are just a few of the conditions listed. Graft-versus-host disease (GVHD) was one of the first conditions to be studied. 

Acute graft-versus-host disease (aGVHD) is a major cause of morbidity and mortality after allogeneic hematopoietic stem cell transplant or donor lymphocyte infusion. This can occur in up to 30–50% of patients despite HLA-matched sibling transplant and even more frequently in HLA-mismatched unrelated donor transplants (60–80%) [23]. Corticosteroids remain the first-line treatment; however, despite the addition of other steroid sparing agents such as calcineurin inhibitors, prognosis for steroid-refractory aGVHD patients remains poor with 5-year survival of less than 30%. Furthermore, many patients may either progress from aGVHD or develop de novo chronic GVHD (cGVHD) with similar high risk of morbidity and mortality [23]. MSC has been examined for use both in the prevention and treatment of acute and chronic GVHD. We review here the clinical trials of MSC in the prevention and treatment of GVHD and discuss some of the challenges that remain to be addressed.

## 2. MSC in the Treatment of Graft-versus-Host Disease

### 2.1. MSC Harvested from Bone Marrow Aspirate for IV Infusion

The first case of MSC infusion for treatment of GVHD was described by Le Blanc et al. in 2004 [24]. A nine-year-old boy with acute lymphoblastic leukemia received a matched unrelated donor peripheral blood stem cell transplant after his third remission. He developed rash on day 11, diarrhea on day 22, and liver enzyme elevation on day 25 after transplant. He did not respond to corticosteroids, extracorporeal photochemotherapy, infliximab, daclizumab, mycophenolate mofetil, and methotrexate. By day 70, aGVHD had progressed to grade IV severity, and on day 73, he received an IV infusion of 2 × 10^6^ cells/kg of MSC from his mother. No adverse events were noted with the infusion, and it was reported that symptoms began to improve within 5 days and resolved within two weeks. The patient developed recurrent GVHD at day 150, shortly after his immunosuppression was discontinued to treat minimal recurrent disease. He received another infusion of MSC from his mother at half of the first dose. He was reported to have a complete response of his GVHD and remission of ALL. He subsequently died 19 months after transplant from recurrence of gut GVHD and respiratory failure from pneumonia [25]. 

This patient was also included in a pilot study reported by Ringden et al. [25] of eight patients with steroid-resistant grade III to IV aGVHD and one patient with chronic GVHD who were treated with MSC between October 2001 and March 2005. Two patients were children, and seven were adults. MSC was cultured from bone marrow aspirates of healthy donors and used fresh from culture at or under four passages. Flow cytometry analysis for phenotype was performed prior to release. Six patients received one infusion, and three patients received two infusions. The median MSC dose was 1 × 10^6^ cells/kg (range 0.7–9). Of the twelve infusions, two were from HLA-identical donors, six from haploidentical donors, and four from mismatched unrelated donors. Two patients who received two MSC infusions had different donors for each infusion. The median time between transplant and MSC infusion was 108 days (range 32–283 days) and between onset of GVHD symptoms and infusion was 37 days (range 7–90 days). There were no adverse events reported from the MSC infusions, and six of the eight aGVHD patients had complete response. The patient with chronic GVHD was reported to have a transient liver response without any skin response. Median response time was not reported. In addition to the pediatric patient previously described in Le Blanc et al. '04, one of the remaining two patients who received two infusions did not have significant response to the first infusion of 0.7 × 10^6^ cells/kg but was reported to have had a complete response with second infusion of 2 × 10^6^ cells/kg. The third patient who received two infusions also had an escalation of dosage from 0.7 to 1.3 × 10^6^ cells/kg. He did not respond to either infusion and died eight days later from multiorgan failure. No patient developed chronic GVHD. 

In autopsy of two nonresponders, HLA-specific DNA from MSC donor was found in the GI lymph nodes of the nonresponder who died eight days after infusion, but not identified in another nonresponder who died three weeks after MSC infusion. In another patient who had a complete response to MSC infusion who died 19 months later from recurrence of GVHD, MSC donor DNA was not found at autopsy [25]. 

Sixteen patients who had grade II–IV GVHD of the gut during the same time period served as a control. Compared to these patients, those who received MSC infusion had almost three-time longer survival (*P* = 0.03) [25]; however it appeared that there was an initial intention to treat these patients with MSC, and they did not receive therapy due to factors such as limited laboratory resources, patient refusal, progressive primary malignancy, and improvement in GVHD. 

This report was followed shortly by a report from Muller et al. [26] of MSC infusion in seven pediatric patients between June 2004 and July 2005. Five of the seven patients received MSC to treat GVHD. Two patients had acute GVHD, and three had chronic GVHD. Two chronic GVHD patients received two MSC infusions. Two patients who received stem cell transplant from matched unrelated donors received MSC from haploidentical family donors. The remaining three patients received MSC from their same HSC donors: one matched sibling donor and two haploidentical family donors. The median time from transplant to infusion was 250 days (range 80–768 days). The median time between onset of symptoms and infusion was not reported. The median dose was 2.5 × 10^6^ cells/kg (0.4–3 × 10^6^). MSCs cultured for less than 6 weeks were used fresh from culture without frozen storage. The variation in dosage was largely due to technical limitation of the interdonor variation in growth kinetics. No adverse events were reported with MSC infusions; however only one patient with aGVHD responded and remained alive and well two years after MSC infusion and three years after transplant at the time of report. The other patient with aGVHD died of relapsed AML with no change in GVHD status. One patient with cGVHD had a slight improvement and remained alive 2.5 years after first MSC infusion and four years after transplant. A second patient with cGVHD died of cGVHD 1 year after MSC infusion, three years from transplant. The third patient with cGVHD did not respond and died from EBV-associated lymphoproliferative disorder 18 months after MSC infusion, 27 months after transplant. aGVHD response did not appear to be dose dependent as one response occurred after a single dose of 0.4 × 10^6^ cells/kg while others did not respond to two to three doses of 2 × 10^6^ cells/kg. In addition phenotype purity information was not available on two patients and low at 83% for one patient. The significance of lower purity is unclear as patients who received high-purity MSC did not necessarily respond better. 

Additionally, in this report, a nine-year-old boy with MDS who had received a second hematopoietic stem cell (HSC) transplant from a haploidentical donor was transfused with 2 × 10^6^ cells/kg of MSC from the same HSC donor at day 17 after his second transplant to prevent graft rejection which was successful, and he remained alive and well three years later. Another patient, a fourteen-year-old girl with T-cell ALL received MSC from the same haploidentical HSC donor for hemophagocytosis. She received three doses of 0.4 × 10^6^ cells/kg nine months after transplant. She had partial response with improvement in her platelet count and decreased degree of hemophagocytosis on bone marrow biopsy. She then went on to receive a second HSC transplant which engrafted well, and she was disease free two years later. 

These preliminary reports were followed by the largest study published to date, a multicenter, phase II study of bone marrow-derived MSC for treatment of steroid-resistant aGVHD from the European Group for Blood and Bone Marrow Transplantation Mesenchymal Stem Cell Consortium [27]. Fifty-five patients were treated between October 2001 and January 2007. Twenty five-patients were children with an overall median age of 22 years (range 0.5–64 yrs). A majority had grade III (*n* = 25) or IV (*n* = 25) GVHD. Nine patients had GVHD involvement of three organs, and twenty-six patients had involvement of two organs. 

The median time from transplant to MSC infusion was 103 days (range 27–533 days) using HLA-identical (*n* = 5), haploidentical (*n* = 18), and HLA-mismatched unrelated (*n* = 69) donors. Most patients in the study received one (*n* = 27) or two infusions (*n* = 22) with a median dose per infusion of 1.4 × 10^6^ (range 0.4–9 × 10^6^) cells/kg. MSC were harvested from culture at 4 passages or less and frozen until use. Of the patients who received more than one infusion, seven patients (6 children, 1 adult) received a second infusion for GVHD prevention when immunosuppressive drug was reduced. Five patients had a complete response initially and received subsequent doses with GVHD recurrence. Seventeen patients without sustained complete response and five patients with partial responses received repeat doses. Of those, 12 patients did not respond despite multiple infusions. 

The overall response rate was 71% (39/55, CR 30, PR 9) with a median time to complete response after infusion of 18 (range 3–63) days. The response rate after one dose was 52.7% (27 CR and 2 PR, *n* = 55). A majority (*n* = 24) of these responders received MSC from HLA-mismatched unrelated donors. There was no difference in the MSC dose between responders and nonresponders. One patient who did not respond to a first dose of 0.6 × 10^6^ cells/kg did respond to a second dose of 2 × 10^6^ cells/kg. The overall response rate was slightly higher in the pediatric population (pediatric: 84%, 21/25; adults: 60%, 18/30; *P* = 0.07). However the study was inadequately powered to demonstrate statistical significance between the two populations. There was no statistically significant correlation between response to MSC infusion and type or severity of GVHD, treatments prior to MSC infusion, age or HLA-matching of MSC donors, or time between onset of GVHD symptoms and MSC infusion. It was noted that variable responses were seen when cells expanded from one donor were given to several recipients. 

Transplantation-related mortality was significantly lower in complete responders compared to partial and nonresponders (100 days: CR 13% versus non-CR 60%, *P* = 0.002; 1 year: CR 37% versus non-CR 72%, *P* = 0.002). With a median followup of 16 months (1.5–64 months), the overall estimated 2-year survival for the patients in this study was 35% (95% CI 22–38%) and was significantly better in complete responders (52%, 95% CI 34–70%) versus noncomplete responders (16%, 95% CI 0–32%, *P* = 0.018). There was also a suggestion of better trend for 2-year estimated survival in pediatric population compared to adults, although the study was inadequately powered to address this. Nine of the MSC responders died from infections. Of the 21 survivors, eight patients developed chronic GVHD, with four having extensive or severe involvement. Three patients had recurrence of their hematologic malignancies (one multiple myeloma, one acute lymphoblastic leukemia, one acute myeloid leukemia). One pediatric patient with Pearson's disease developed acute myeloid leukemia de novo from endogenous hematopoietic cells. One patient developed Epstein-Barr virus-associated posttransplant lymphoproliferative disorder. 

This landmark study was the first large-sample, prospective, multicentered study with a longer median followup that suggested that MSC therapy may be effective in treating GVHD. The use of different types of MSC donors suggested that clinical responses could be achieved with third party unmatched MSC raising the possibility of storage for immediate access.

### 2.2. Clinical Trials Exploring Other Methods of MSC Culture and Deliveries

Arima et al. [28] reported a limited series of three adult patients who were treated with intra-arterial infusion of MSC for localized treatment of liver and gut GVHD between April 2006 and May 2008. These patients were steroid refractory with either a calcineurin inhibitor or mycophenolate mofetil. Fresh MSCs harvested from bone marrow aspirates of their respective HSC donors were cultured in donor serum up to three weeks and then infused into superior mesenteric, gastroduodenal, and inferior mesenteric arteries for gut GVHD and common hepatic artery for hepatic GVHD via selective angiography. No immediate adverse events were associated with the MSC infusions. The dose of MSC was significantly limited by the rate of culture expansion and was significantly less than other studies (median 0.51 × 10^6^ cells/kg/treatment, range 0.32–2.0 × 10^6^ cells/kg/treatment, in fractions of 0.08–0.50 × 10^6^ cells/kg/arterial distribution). Unfortunately, no major response was observed. One patient had overall stability of GVHD and died of respiratory failure from idiopathic pneumonia 103 days after MSC infusion (199 days after transplant). A second patient had a transient improvement in gut GVHD two weeks after MSC infusion as proven by biopsy. He had recurrence of GVHD five weeks later and died from intestinal obstruction and hepatic failure 58 days after MSC infusion, day 178 from transplant. The third patient had progressive GVHD and died from respiratory failure 20 days after MSC infusion, day 178 from transplant. On autopsy, she was found to have hepatic GVHD, diffuse alveolar damage of the lung without evidence of GVHD involvement or infection, and severe CMV and EBV colitis which interfered with accurate assessment of GI GVHD involvement. The study was stopped due to lack of GVHD response. At least in this study, donor serum did not appear to improve the culture yield of MSC and added to the complexity of the manufacturing process. Subsequent study using platelet lysate reported below may be a more accessible method of manufacturing. Although this study was limited to three patients, it did not appear that direct injection of MSC to site of GVHD involvement improved the treatment response, leading to the larger question of homing pattern and mechanisms of actions of MSC *in vivo*. At any rate, given the added risk of invasive angiography without additional benefit, this method was not pursued further. Finally the findings of idiopathic pneumonia and severe viral enteritis raise the concern of MSC-exacerbating risk and severity of infection in this already immunocompromised population.

Zhou et al. [29] reported four adult patients with chronic sclerodermatous GVHD who received MSC between September 2006 to August 2008. The MSC infusions were given directly into the bone marrow via anterosuperior iliac spine. The MSCs used were up to 6 passages in culture, one of the highest reported in the literature. The immunophenotype was slightly different from others (no CD73). Multilineage differentiation was confirmed in culture. The MSC infusion was limited to 1-2 × 10^7^ cells per infusion, which would be significantly less than the average 1-2 × 10^6^ cells/kg in the other studies. The patients did receive 4 to 8 repeated infusions, which is the highest number of repeat infusions examined. Again, no immediate adverse events were observed with MSC infusions. All four patients were reported to have had significant improvement in their Rodnan Skin score (>70% improvement) and improved joint mobility. One patient had significant tapering of GVHD medications. With a median followup of 14.1 (4.6–23) months, all patients were alive. None had recurrent primary hematologic malignancies. In addition, correlative studies showed that in all four patients, there was a consistent trend in all patients with an increased ratio of Th1 to Th2 cell ratio as treatment continued. Although the dosage of MSC was less than that used in previous aGVHD study, the smaller doses with multiple infusions appears to have promise in cGVHD raising the question of frequency and timing rather than just dose as a factor in response. Whether intra-BM infusion truly bypasses lung sequestration and improves efficacy by increasing MSC in the affected microenvironment requires further study. This method of infusion is certainly more cumbersome and less desirable if IV infusion is equally efficacious. 

In followup of the Arima et al. study that used human serum to culture bone-marrow-derived MSC, Perez-Simon et al. [30] reported a series of 18 adult patients treated with MSC cultured with human serum. In feasibility assessment of the manufacturing, 28 MSC expansions were attempted. 78.5% (22/28) of the cultures achieved the goal of >1 × 10^6^ cells/kg with a median of 26 days of culture. Notably, in eight cultures, platelet lysate was also added to improve the growth of MSC. In only one instance was MSC treatment not given due to inability of full expansion of MSC despite adding platelet lysate. Of the eighteen patients treated between February 2007 and December 2009, 10 patients had aGVHD, 8 had cGVHD. Patients received 0.2–2.9 × 10^6^ cells/kg per dose up to four doses. In aGVHD, five patients received one dose, one patient each received 2 and 3 doses, and three patients received four doses. None of the patients who received more than one dose had a better quality of response. One patient had CR, 6 had PR, and 3 did not respond. At the time of publication (last FU median FU not given), only one patient was alive. Among the nine who have died, two died from GVHD, five from infection, one from relapsed disease, and one from venoocclusive disease. In cGVHD, four patients received one dose of MSC, three received two doses, and one patient received three doses. One patient had CR, 3 PR, and 4 did not respond. The patient who had CR also had severe thrombocytopenia which resolved after MSC infusion. This data combined with 4 pts in Zhou et al. support additional study of this modality in adult cGVHD setting.

Given the concern for possible prion disease with fetal bovine serum, von Bonin et al. [31] also reported the use of MSC expanded in media containing human platelet lysate for the treatment of steroid-refractory aGVHD in their compassionate use program. Bone marrow aspirates from 12 healthy donors were expanded for one or two passages with media containing 10% platelet lysate derived from single-donor platelet apheresis concentrate. The MSC were examined by flow cytometry for phenotype, their osteogenic and adipogenic differentiation capacity were tested by standard differentiation culture, and their immunosuppressive capacity was tested via suppression of CD4^+^ T cell proliferation by thymidine incorporation assay. The MSCs were not HLA matched to aGVHD patients. Thirteen patients were recruited between March and December of 2007. All patients had failed steroid plus another immunosuppressive agent. The median time from transplant to first MSC infusion was 41 days (range 20–91) and from onset of aGVHD was 16 days (range 4–31). The median number of MSC infusions was 2 (range 1–5). The median interval between infusions was 7 days (range 3–19). The median dose given each time was 0.9 × 10^6^ cells/kg (range 0.6–1.1 × 10^6^ cells/kg). There were no adverse events with infusions. At 28 days after infusion, there were one CR, one PR, and five MR. For each of the affected organs, the response rates were 3/6 CR and 0/6 PR for skin, 2/10 CR and 3/10 PR for liver, and 2/11 CR and 2/11 PR for GI. At the time of report, 9 patients have died: four within 28 days after MSC infusion and four during the two years of followup. Four were alive with a median followup of 257 days (range 185–692) from transplant. The authors reported that the 1- and 2-year survivals of patients with similar grades of steroid-refractory GVHD at their institution who did not receive MSC were 28% (11/40) and 20% (8/40), respectively. While no formal statistical analysis was performed, the survival appears to be comparable. The small number of patients in this report makes assessment difficult regarding intersample variability between MSC from different donors, as well as fresh versus frozen MSC and MSC functional capacity variation with different platelet lysate. Nevertheless platelet lysate may be a more readily available human blood product for MSC culture. 

Fang et al. [32] had the only report of adipose-derived MSC in the treatment for steroid-refractory aGVHD. Six patients were recruited between September 2002 and August 2005. Adipose-derived MSCs were harvested from abdominal adipose tissue of donors undergoing lipectomy surgery. Cells were assessed for phenotype by flow cytometry prior to release. The median time of first MSC infusion from transplant was 71 days (range 65–243) and from onset of GVHD was 38.5 days (range 7–58). MSC dose was 1 × 10^6^ cells/kg. Overall, five of the six patients had complete response (83.3%). One of the five responders died 13 months later from relapsed AML. The remaining four were alive and well at the time of report, with median survival after transplant of 26.5 months (range 18–90 months). One patient who had GVHD of skin, liver, and GI had partial response with first infusion. She had progressive symptoms 47 days later and received another MSC infusion with complete response. The patient who did not respond to MSC infusion had skin and GI GVHD. She died 16 days after MSC infusion from multiorgan failure after systemic fungal infection and hemorrhagic cystitis. Larger sample study is warranted to assess the efficacy of this more readily available source of MSC for this clinical application.

### 2.3. Commercially Manufactured MSC (Prochymal)

In the largest, prospective, open-labeled multicentered phase II study conducted in the US, Kebriaei et al. [33] randomized adult patients with de novo aGVHD to IV MSC infusions at either 2 × 10^6^ or 8 × 10^6^ cells/kg along with corticosteroid. Thirty one patients were treated among 16 centers between April 2005 and June 2006. The MSC, Prochymal, was manufactured by Osiris Therapeutics Inc. from bone marrow aspirates of 6 healthy donors, cultured in fetal bovine serum, and frozen until IV infusion. Lot-to-lot consistency was ensured by immunophenotyping via flow cytometry (per International Society for Cellular Therapy position statement), documented cell viability, functional testing by TNFR1 expression quantification on MSC, and qualitative assay measuring the inhibition of IL2Ralpha expression on activated T cells by MSC. Sixteen patients received low-dose MSC, and fifteen patients received high dose. Each patient received MSC from one donor. Because of improved MSC availability with premanufacturing, all patients received their first MSC infusion within 48 hours from diagnosis of grade II–IV GVHD and a second infusion 3 days later. Of note, more than half of the patients had grade II GVHD which was lower severity than the Le Blanc '08 study (10/15 high-dose group and 11 of 16 low-dose group). No adverse events were seen with MSC infusions. No ectopic tissue formation was seen with radiologic imaging. Initial response rate was high, with 24 CR (10 in high-dose group, 14 in low-dose group) and 5 PR (high dose group). 19 patients who achieved CR did not require second-line treatment in 90 days. Time to response was also rapid with 42% patients achieving CR at day 7, 52% by day 14, and 77% at day 28. CR was not correlated to donor source, GVHD location, or grade. 22/31 patients survived to 90 days. Patients who achieved CR had significantly improved survival than non-CR patients (CR: 21/24, 88%; non-CR: 1/7, 14%; *P* = 0.0008). Three patients who had achieved CR died from infections (pneumonia, meningitis, aspergillus enteritis). Median time to death for the nine patients was 44 days from MSC infusion (13–63 days). Those in non-PR died from progressive GVHD (3), relapsed malignancy (1), and CNS bleed (1). In terms of risk of primary malignancy relapse, three patients relapsed in a two-year follow-up period. Twelve grade 3 infections and three grade 4 infections, per CTC version 3, were seen. This is the first large prospective study of MSC therapy in de novo aGVHD. It also confirmed the feasibility of commercial manufacturing, allowing some uniformity in release criteria and more immediate accessibility. The good clinical response may be due to both early initiation of MSC therapy from onset of aGVHD and a higher proportion of patients with lower-grade severity. Results suggest better response in patients with lower GI GVHD. This may be consistent with autopsy finding from previous study of MSC homing to GI lymph node within days after infusion [25].

Prasad et al. [34] reported the use of Prochymal in pediatric patients with grade III/IV refractory aGVHD. Twelve patients were treated between July 2005 and June 2007. The median number of therapies other than steroid was 3 (range 2–5). MSCs premanufactured from four healthy adult donors were used. The dosing schedule was twice weekly infusion at least 3 days apart for weeks (induction phase). For patients who had partial response or mixed response at 32 days evaluation, weekly infusion at the same cell dose was given for 4 more weeks (maintenance phase). Those who had achieved complete response or no response at 32 days evaluation did not receive additional infusions. The first two patients were treated with a higher dose of 8 × 10^6^ cells/kg before protocol amendment to reduce the dose to 2 × 10^6^ cells/kg for the remaining 10 patients on the study. The protocol was amended due to finding of equivalent efficacy with both doses from Kebriaei et al. The median duration between transplant and MSC therapy was 98 days (45–237) and between aGVHD diagnosis and MSC therapy was 46 days (18–157). The median number of infusions per patient was 8 (2–21). By the end of induction phase, two patients achieved CR, and 6 had PR. By the end of all treatments, 7 patients (58%) had CR, two PR (17%). Two patients who achieved CR had recurrence of grade IV skin and gut GVHD, respectively. They were retreated with MSC infusion with significant PR, with symptom improvement to grade I. All 12 patients had gut GVHD. The CR response rate to gut involvement was 75% (9/12). Survival at 100 days was 58% (7/12). Five of 12 (42%) patients were alive after a median followup of 611 days (427–1111). The estimated 2-year survival for patients treated with MSC was 40% (95% CI: 20–82%) and for patients achieving CR was 68% (95% CI: 40–100%). The median time to death from MSC infusion was 58 days (36–185 days, *n* = 7). Two patients with CR died from sepsis (stenotrophomonas and pseudomonas aeruginosa). Five non-CR patients died from infections (*n* = 3, fungal *n* = 2, CMV encephalitis *n* = 1), EBV-associated lymphoproliferative disorder (*n* = 1), and multiorgan system failure (*n* = 1).

Osiris Therapeutics Inc. had two phase III trials investigating their product Prochymal in first-line treatment and in steroid-refractory aGVHD across transplant centers in United States, Canada, and Australia. The trial in first-line aGVHD was a double-blinded, placebo-controlled trial that enrolled 192 patients. Primary endpoint was the proportion of patients surviving at least 90 days with a complete response in steroid alone versus those who received steroid along with Prochymal. Treatment failure was defined as failure to achieve complete response in 28 days, need to increase steroid dose, or addition of other immunosuppressive agents. Accrual for both studies is complete. No peer-reviewed publication of trial results is available yet. The last company released news report in 2009 did not show a statistically significant difference (Prochymal 45% versus placebo 46%, *n* = 192, http://www.osiristx.com/). 

In the steroid-refractory population, the primary endpoint in this phase III, double-blind, placebo-controlled study was durable complete response for at least 28 days. Patients were randomized at 2 : 1 ratio for either Prochymal or placebo. This trial enrolled 260 patients. In the company's public news release in 2009, the preliminary analysis has not shown a statistical difference between Prochymal and placebo on primary endpoint in this population (Prochymal 35% versus placebo 30%, *n* = 260). However subpopulation analysis did show an improved response rate in liver GVHD (Prochymal 76% versus placebo 47%, *P* = 0.026, *n* = 61) and GI GVHD (Prochymal 88% versus placebo 64%, *P* = 0.018, *n* = 71). Patients with liver GVHD also had significant durable complete response with Prochymal (Prochymal 25% versus placebo 5%, *P* = 0.046). The company reasoned that the lack of statistical significance in the overall population may be related to the higher response rate already achievable with standard of care in the skin GVHD patients, which is a significant proportion of the accrued patients. 

In addition, consistent with previous literature reports, the combined pediatric cohort from both studies showed strong trend in improvement of response rate (Prochymal 86% versus placebo 57%, *P* = 0.094, *n* = 28). Based on this subgroup analysis, Osiris filed an amendment with the FDA to broaden the entry criteria to include patients with severe GVHD of the liver. In addition FDA has granted expanded access, making Prochymal available to children with GVHD.

### 2.4. Summary

In summary, clinical data to date support the safety of MSC to treat acute and chronic GVHD (summarized in Tables 1 and 2). Multiple early phase studies have explored the feasibility of different methods of MSC manufacturing and delivery. While phase II studies suggest clinical efficacy of this modality, two large, multicenter, prospective phase III trials have examined the use of MSC to treat de novo aGVHD and therapy-refractory acute and chronic GVHD without evidence of efficacy as determined by the primary endpoints. Better understanding of the mechanism of action of this cell therapy modality is needed to optimize therapy and identify the GVHD population that may benefit from this treatment. 

The largest clinical experience has been with IV infusion of MSC cultured from bone marrow-aspirate in fetal bovine serum. Some important factors have helped ease the transition to commercialize the manufacturing process of this treatment modality. First, the finding of comparable efficacy and lack of toxicity in MSC from HLA-mismatched unrelated donors allows the use of premanufactured, cryopreserved MSC from a larger donor source, thereby removing the time constraint and increasing the accessibility of MSC. Second, commercially available, quality-controlled culture media and supplement can help minimize some of the intersample variability in the culture process. To further improve accessibility and reduce cost, the utility of other more readily available sources of MSC such as adipose tissue warrants further study. In addition, platelet lysate may be another viable alternative for culture supplement to improve growth kinetics while reducing concern for zoonosis as a concern with animal serum. The current phenotype criteria to characterize MSC in general does not identify phenotype variation within this heterogeneous population of cells that may make a batch of MSC from one donor more effective from another. Identification of such phenotype could improve the efficiency of this technology. 

The concern for increased risk of disease relapse and infection due to the immunosuppressive properties of MSC cannot be adequately addressed with the existing data. Heterogeneous patient populations were enrolled across these studies with limited controls for comparison. Many enrolled patients are at inherently at high risk for disease recurrence and infection due to the nature of their disease and treatment. This is a valid concern that will need to be carefully analyzed in randomized, placebo-controlled phase III studies. 

The dose and frequency of MSC infusion to maximize clinical efficacy has not been addressed in these smaller studies. While the technical limitations from manufacturing have improved to allow for infusion in the range of 10^6^ cells/kg, the biokinetics of the infused MSC is poorly understood. The most commonly used dose has been 1-2 × 10^6^ cells/kg. Limited observations suggest that higher dose is not more effective. While some patients did not respond to lower doses, there are reports of those who may even respond to a single dose as low as 0.4 × 10^6^ cells/kg. In addition, the average time to response after infusion appears to be between 1 to 3 weeks [27, 29]. While some patients who respond to first treatment will continue to respond at GVHD relapse, there are those who do not respond to repeated infusion if they did not have response to first infusion. Relevant to finding the optimal dose and frequency is better understanding of MSC trafficking and homing pattern in patients and their mechanism of action in the treatment of GVHD. Donor MSC has not been identified in patients from bone marrow aspirate cultures irrespective of clinical response, suggesting a lack of donor MSC engraftment. Donor MSC has been found on autopsy in area of tissue inflammation such as GI lymph node within days after infusion, but not seen weeks after infusion. The question remains to be addressed whether MSC residence is necessary for its proposed biologic functions. One possibility is that MSC may differentiate into a progeny cell population in the body. Labeling MSC to better track its homing pattern and its fate after homing can address this possibility [35]. MSC also may alter homeostatic microenvironment through its interaction with immune and hematopoietic cells. This effect may persist beyond time of MSC residence in the tissue. Parekkadan and Milwid [36] explored using chemical engineering modeling principles to develop dosing regimen based on this kinetic information.

Data also raise the possibility that subpopulations of patients such as those with specific organ involvement may be more likely to respond to MSC treatment. In the era of personalized medicine, we need more precise methods to identify patients who will likely benefit from this labor-intensive and costly treatment. Clinically relevant prognostic tests and biomarkers that identify those patients who will likely respond to MSC infusion and to assist in decisions in treatment dose and schedule would be very useful but are currently poorly defined. The correlation between changes in ratio of Th1/Th2 cells and clinical response in Zhou et al. study [29] suggest the possibility that such biomarkers may be discoverable. Bioinformatics approaches can be used to tease out the intricacies of this complex patient population to identify distinctive profiles of GVHD patients who may benefit from this treatment modality.

## 3. MSC in the Prevention of Graft-versus-Host Disease

In some animal models, MSC infusion improved the survival of skin and solid organ grafts [4, 16, 19, 21]. Some patients in previously described studies who were at risk of graft failure or rejection had good response with MSC infusion [26]. This prompted a number of investigators to examine the use of MSC cotransplant with HSCT to promote engraftment and prevent GVHD.

### 3.1. MSC Cotransplant in HLA-Identical HSCT

Lee et al. [37] from Korea first reported cotransplantation of allogeneic MSC with HSC in an attempt to improve engraftment. A twenty-year-old woman with high-risk acute myelogenous leukemia (M1 with complex chromosomal abnormalities) received peripheral blood mobilized, T-cell depleted HSC from her haploidentical father. Antithymocyte globulin (15 mg/kg/d) was given as part of her conditioning regimen on day −6 to −3. Five weeks prior to scheduled HSC transplant, MSC was cultured from bone marrow aspirate of her father and harvested after four passages for fresh infusion. She received 1.5 × 10^6^ cells/kg of MSC one hour after the second HSC infusion on day 2 of transplant (total 5.7 × 10^6^ HSC infused over two days). No additional prophylactic immunosuppressive treatment was given after transplant. She received G-CSF from day 4 to 14. Her ANC was above 0.5 × 10^9^ cells/L on day 12, and her platelet was above 20 × 10^9^/L without transfusion support by day 15. Bone marrow and peripheral blood mononuclear cell evaluation at day 18 showed 20–30% cellularity in the bone marrow and trilineage engraftment and complete donor chimerism by PCR. On day 42, she had an episode of CMV pneumonia that was successfully treated with ganciclovir and IV immunoglobulin. She maintained engraftment for up to 31 months at the time of report, did not develop acute or chronic GVHD, and remained well without signs of AML relapse. Interestingly, culture of her bone marrow-derived MSC at six and 12 months after transplant showed host genotype despite maintenance of donor genotype in hematopoetic mononuclear cells. 

With this encouraging report, a number of investigators have examined the role of MSC in cotransplantation. Lazarus et al. [38] reported a phase I study across seven centers in the U.S. and Italy. Fifty-six adult patients were enrolled between December 1999 and March 2001. MSCs were cultured from bone marrow aspirate of their respective HLA-identical HSC sibling donors. All samples were transported to production center at Osiris Therapeutics, Inc in Baltimore, Md. MSCs were cultured with fetal bovine serum and cryopreserved until use. The planned MSC dosage was 1, 2.5, and 5 × 10^6^ cells/kg, with 18 patients per dosage group. Only MSC cultures from five donors were able to achieve the highest dosage. Treatment arms were adjusted to the two lower dosages for subsequent patients. MSC culture was feasible in 91% of cases (51/56). The median time to achieve the intended dosage was comparable for the different dosages, 30 days (range 21–48 days). A total of 46 patients were treated with MSC infusions (1 × 10^6^ cells/kg *n* = 20, 2.5 × 10^6^ cells/kg *n* = 21, 5 × 10^6^ cells/kg *n* = 5) followed by HSC infusion four hours later (BM HSC *n* = 19, PB HSC *n* = 27). All patients received GVHD prophylaxis with IV cyclosporine (3 mg/kg/d) starting at day 1 that was adjusted to oral doses as deemed clinically appropriate with discontinuation by 6 months after transplant. Methotrexate was also given IV on days 1, 3, and 6 (10 mg/m^2^). Day 11 methotrexate was eliminated due to concern for potential toxicity to infused MSC. While all patients experienced at least one adverse event as expected associated with HSCT during the peritransplant period, no immediate toxicity was noted with MSC infusion, and no ectopic tissue formation was noted on radiologic imaging as part of routine clinical care. Recombinant hematopoietic growth factor support was not routinely given. The median time to engraftment appeared to be comparable across dosage groups: median time to ANC > 0.5 × 10^9^/L 14 days (11–26), ANC > 1 × 10^9^/L 15 days (12–24), platelet > 20 × 10^9^/L 20.5 days (15–36), and platelet > 50 × 10^6^/L 21 days (15–36). The ANC engraftment was slower for those who received BM HSC which is consistent with general population of patients who receive BM versus PB HSC. Twenty-three patients (50%) experienced aGVHD, with 5 (11%) patients having grade III severity, and 2 (4%) at grade IV. Of the thirty-six patients who were evaluable past 90 days after transplant, twenty-two patients (61%) developed cGVHD (limited 14, extensive 8). The incidence was higher in patients who received BM HSC versus PB HSC. Twelve patients had relapse or progression of their primary hematologic malignancy with a median time of 214 days following transplant (14–688). The estimated 2-year progression free survival and overall survival for the 46 treated patients were 53% (95% CI: 37–66%) and 78% (95% CI: 63–87%), respectively. The causes of deaths were infection (2), relapse (2), hemorrhage (2), hepatic venoocclusive disease (1), GVHD (1), cardiac (1), and GI (1) dysfunction. At a median time of 8 months (*n* = 18) and sixteen months (*n* = 7) following transplant, patients underwent bone marrow aspirate evaluation for MSC chimerism. Only two patients were found to have persistent donor chimerism in their cultured MSC at less than 14%. The overall conclusion from this study is that cotransplantation is technically feasible (>90% manufacturing success) and safe. 

Ball et al. [39] also reported at that time a phase I/II study of 14 children treated with MSC cotransplant along with haploidentical HSC from relatives. MSC culture and HSC collection were successful in all donors. MSCs at less than 3 passages were used either fresh or after cryopreservation. Mean MSC dose was 1.6 × 10^6^ (1–3.3 × 10^6^) cells/kg. No toxicity was seen with MSC infusion. Compared to the 47 historical controls from the two participating institutions, the 14 patients who received cotransplant had a faster engraftment: leukocyte > 1 × 10^9^/L, median 11.5 days (range 9–15 days) for study patients versus 14.9 days (10–26) for controls, *P* = 0.009; reticulocyte > 20 × 10^9^/L, 12 (10–31) days versus 23 (9–41) days, *P* = 0.03. Although ANC and platelet recovery trend was also earlier by a few days, this did not reach statistical significance. The faster rate of leukocyte engraftment was attributed to lymphocyte recovery. These patients also had sustained engraftment (0% failure versus 15% in controls, *P* = 0.14) and no increased risk of infection. Two (14%) patients developed aGVHD of grade I-II severity, and one (7%) patient developed limited involvement cGVHD. This was lower than historical control although statistical significance was not reached. Chimerism analysis of bone marrow-derived MSC from recipient at 3 months interval up to one year did not identify significant donor chimerism. Only three patients had a transient donor MSC engraftment at less than 2% at 3 months. Four patients died (28%) compared to 11 (40.7%) in the control group. The estimated relapse rate and overall survival were not comparable between the two groups due to difference in followup. Among the study patients, two died from relapsed disease and two from infection. With a median followup of 9 (2–28) months, the estimated relapse rate was 18%, and overall survival was 72%. 

Ning et al. [40] reported their single-center experience in China with the highest rate of adverse events. In this open-label trial for patients undergoing HLA-identical sibling BM, PB, or both HSC, patients were randomized to receive either HSC per standard transplant protocol or HSC cotransplant with MSC from the same HSC donor. Thirty patients were enrolled between March 2003 and March 2004. There was one 17-year-old patient in the MSC cotransplant group and one 16-year-old patient in the control group. The remainder of the patients were all adults. Characteristics of the primary hematologic malignancy were comparable between the groups. Conditioning regimen was equivalent between the groups. Cyclosporin and methotrexate (day 1, 3, 6) were used for GVHD prophylaxis. The planned dose of MSC for infusion was 1-2 × 10^6^ cells/kg in 30 days. This was achieved in only two patients. Therefore the MSC infusion dose was reduced, and five enrolled patients were still excluded from the protocol due to inability to meet MSC production criteria. The feasibility of manufacturing in this study was 66.7% (10/15). The median MSC dose infused was 0.34 × 10^6^ cells/kg (0.03–1.53 × 10^6^ cells/kg). No immediate toxicities were observed with MSC infusion. The engraftment time was not statistically significantly different between the two groups. One patient who received 0.34 × 10^6^ cells/kg of MSC in cotransplant did not engraft and died 19 days after transplant. With MSC cotransplant, both the incidence and severity of GVHD were decreased compared to the control group. Four of nine (44.4%) patients in MSC group had aGVHD, while 11/15 (73.3%) patients in the control group had aGVHD. Only 1 (11%) patient in MSC group had grade II aGVHD while 8 (53.3%) did in the control group. One (of seven, 14.3%) patient in the MSC group had limited cGVHD while four (of 14, 28.6%) patients in the control group had cGVHD, 1 limited, and 3 extensive. The rate of infection was comparable between the two groups, four of 10 in MSC and 5 of 15 in control. However, the MSC cotransplant group had significantly higher rate of disease relapse at an earlier time than the control group (MSC: 6/10, 60%, median time 63 days, range 39–1062 days; control: 3/15, 20%, median time 177 days, range 66–450 days; *P* = 0.02). With a median followup of 36.6 months (0.6–44 months), six patients in the MSC group and five in the control group have died. The three-year disease-free survival was 30.0% for MSC and 66.7% for control group (*P* = 0.035). The three-year overall survival was 40.0% for MSC and 66.7% for control group. Rate of GVHD and relapse did not correlate with MSC dose. The investigators closed the study earlier than the planned 25 patients per arm due to this observation of increased disease relapse. 

Zhang et al. [41] reported the results of MSC cotransplantation with HLA-identical sibling PBSCT in a single center in China. Fourteen patients were enrolled between February 2002 and September 2005. Two months prior to transplant, MSC was cultured from the bone marrow aspirate of the same HSC donor. Commercially available culture media supplement (Human MSCs Stimulatory Supplements, StemCell Technologies) was used. MSC were cryopreserved until transplant. Two (14.2%) of the fourteen cultures were unsuccessful. Culture time for the remainder of the samples was 36.8 ± 4.5 days. Twelve patients subsequently underwent cotransplantation. MSC were infused 1 hour after HSC (1.78 ± 0.35 × 10^6^ cells/kg). Cyclosporine and methotrexate (15 mg/m^2^ on day 1, 10 mg/m^2^ on day 3, 6, 11) were used for GVHD prevention. Median time to granulocyte engraftment (>0.5 × 10^9^/L) was 11 days (7–15), platelet engraftment (>20 × 10^9^/L) was 13.5 (10–17) days. All patients had 100% donor chimerism in their peripheral blood mononuclear cells 1 month after transplant. Three patients did not develop aGVHD. Seven had grade I aGVHD. Three of these patients progressed to cGVHD, two limited, and one extensive. Two patients developed grade II-III aGVHD, one of whom progressed to extensive cGVHD. The two patients with extensive cGVHD were treated with cyclosporine and steroid. One responded to treatment, and the second patient died from infection. Four patients had CMV infections, all responded to ganciclovir and IV immunoglobulin. Four (33.3%) patients had disease relapse. They received DLI, and only one patient responded. The five deaths were due to relapse (3) and infections (2, pneumonia and liver failure from hepatitis B). The remaining seven patients were alive 29–57 months after transplant at the time of report.

### 3.2. MSC Cotransplant in Other Types of HSCT

Le Blanc et al. [42] reported a small pilot study in Europe where seven patients (3 adults and 4 pediatric) received MSC cotransplant with HSC. This was a second transplant for three patients in the study, two for graft failure, and one for graft rejection. The HSC sources were PBSC (4), BM (2) and cord blood (1). HSC donors were matched sibling (3), matched unrelated donor (2), and mismatched unrelated donors (2). MSC donors were the same as HSC donors for the matched sibling and haploidentical donors for those whose HSC donors were unrelated. All MSCs were cultured from bone marrow aspirates of adults in fetal bovine serum. All patients received 1 × 10^6^ cells/kg of MSC within 4 hours of HSC infusions. Six patients received thymoglobulin prior to HSCT. One patient developed anaphylaxis to thymoglobulin and received Campath instead. For GVHD prophylaxis peritransplant, three patients received methotrexate and cyclosporine, two received cyclosporine alone, one with prednisolone and cyclosporine, and one with tacrolimus and sirolimus. Hematopoietic growth factor support was not given. Median engraftment time for ANC > 0.5 × 10^6^/L was 12 (10–28) days, for platelet > 50 × 10^6^/L was 15 (11–45) days. This was on average about a week shorter than the institutional average at that time. In addition, transfusion requirement was low before engraftment, particularly for adults (RBC median 2 (0–6) units, platelet 1 (1–3) units). All three patients who received retransplant due to graft failure or rejection had successful engraftment. No immediate adverse event was seen with MSC infusion, nor was ectopic tissue formation noted. One patient was alive and well 15 months after transplant without GVHD. Four patients developed grade I GVHD, and two patients developed grade II GVHD. These mild GVHD responded to treatment with steroid. Only one patient with grade II GVHD progressed to chronic GVHD of the skin and gut. His symptoms were managed with tacrolimus and steroid, and he was alive and well 28 months following transplant. One patient died from aspergillosis 8 months after transplant. All other patients were alive and well with a median followup of 23.5 (10–37) months after transplant. One patient had serious multibacterial infection within the first month following transplant and one patient had recurrent, serious infections, including Listeria meningitis eight months after transplant. One patient developed VOD and two had mild-to-moderate hemorrhagic cystitis. Even though majority (6/7) of the patients in this study developed GVHD, they were milder, adequately managed, and did not lead to significant morbidity or any mortality. In addition, MSC therapy appeared to be effective in retransplantation with prior history of graft rejection/failure. 

Gonzalo-Daganzo et al. [43] reported a small study that was also stopped early, examining the use of MSC cotransplantation in patients who receive single-unit cord blood and T-cell depleted mobilized PBSC in enhancing engraftment and preventing GVHD. In this phase I/II study, nine adults were treated with MSC cotransplant, and 46 patients treated at the same time in the institution served as control. Baseline patient characteristics were comparable between the two groups. MSC were cultured from bone marrow aspirate of HSC donors 4–6 weeks prior to CB transplant. MSCs were harvested and cryopreserved with a median culture time of 28 days (16–34) and 2 passages (1–3). All CB, PBSC, and MSC were infused within 24 hours. The median dose of MSC was 1.2 (1.04–2.22) × 10^6^ cells/kg. No immediate toxicities were seen with MSC infusion. Cyclosporine and methylprednisolone (1 mg/kg, day 1 to 10–14) were used for GVHD prophylaxis. G-CSF was given on day 1 until ANC was greater than 1 × 10^9^/L. No significant difference was seen in ANC and platelet engraftment or time to 100% CB chimerism. Chimerism analysis at day 15, 35, 100, and one year after transplant for seven patients did not show any donor chimerism. In the MSC group, one (11.1%) patient developed grade I aGVHD, 4 (44.4%) with grade II, and none had grade III-IV. In contrast, 18 (39%) patients in the control group had grade I aGVHD, 5 (11%) had grade II, and 6 (13%) had grade III-IV aGVHD. Among the 4 patients who had grade II aGVHD in the MSC cotransplant group, two did not respond to steroid therapy. These two patients received one and three infusions of MSC and achieved CR. One patient in the MSC group died at day 30 with multiorgan system failure without aGVHD. The remaining patients were alive with median followup of 8 months (3.5–22). There was one (11.1%) disease relapse in the MSC group and six (13%) in the control group. Two-year cumulative incidence of transplant-related mortality was 0.11 (95% CI: 0.02–0.71) for MSC group and 0.37 (95% CI: 0.25–0.54). While the trend in decreased TRM and increased OS is interestingly, the study was stopped early, and the sample size was too small to assess for statistical significance. The investigators reasoned that the dual single-unit CB and PBSC transplant morbidity and mortality were better than they had predicted. Therefore MSC cotransplant in this setting may not be cost-effective. In addition their observation of effectiveness of MSC infusion at the time of steroid-refractory aGVHD along with others reported shifted their practice to design studies investigating the role of MSC infusion in treatment rather than prophylaxis of GVHD in this type of dual transplant. 

The most recent publication by Baron et al. [44] reported cotransplantation of MSC in the higher risk population of patients receiving HSC from HLA-mismatched unrelated donor with nonmyeloablative conditioning regimen. Twenty adult patients enrolled between January 2007 and September 2008 received cotransplant with HSC from HLA-mismatched unrelated donor and MSC from third-party unrelated donors. Sixteen patients with similar baseline characteristics who received similar HSC transplant between May 2002 and August 2006 served as control. MSCs were harvested from bone marrow aspirate and cultured for 2 passages, then cryopreserved until use. MSC cotransplant patients received conditioning regimen with Flu (30 mg/m^2^, days −4, −3, −2) and TBI (2 Gy, day 0). MSCs were infused between 30–120 minutes before HSC infusion. GVHD prophylaxis included mycophenolate mofetil (15 mg/kg po TID, day 0–42) and tacrolimus (titrated to keep blood levels 15–20 ng/mL through day 28 and 10–15 ng/mL thereafter, po day −3 to be tapered off by one year if no symptoms of GVHD). The engraftment time was not significantly different between the two groups. The proportion of patients who developed severe aGVHD were lower with MSC cotransplantation, although this did not reach statistical significance (grade II–IV: 45% versus 56%; grade IV: *n* = 2, 10% versus *n* = 3, 19%). However the one-year probability of dying from GVHD or infection while on treatment for GVHD was significantly lower with MSC cotransplant (10% versus 31%, *P* = 0.02). In addition the 100-day (5%) and 1-year nonrelapse mortality were significantly lower with MSC cotransplant (1 year: 10% versus 37% in control group, *P* = 0.02). The one year overall survival was significantly improved with MSC cotransplant (80% versus 44%, *P* = 0.02). In multivariate analysis, MSC cotransplant was strongly associated with reduced NRM (HR = 0.2, 95% CI: 0.04–0.9, *P* = 0.03) and overall mortality (HR = 0.4, 95% CI: 0.1–0.9, *P* = 0.03). In contrast to the Ning et al. report earlier, these investigators did not find decreased GVT effect as evidenced by equivalent 1-year cumulative incidence of disease relapse and equivalent disease CR rate after transplant in patients who had residual disease at the time of transplant. Consistent with prior studies, the incidence of grade IV aGVHD was lower with MSC cotransplant. This is the first study to confirm survival advantage of MSC cotransplant by multivariate analysis. A large, multicenter study is underway in Belgium to determine if these findings will be confirmed.

### 3.3. Summary

Similar to MSC therapy for treatment of GVHD, MSC cotransplant with HSCT appears to be safe with minimal infusion-associated toxicities across multiple studies (summarized in Table 3). Preliminary studies do not suggest dramatic improvement in the rate of HSC engraftment with cotransplantation. However there may be a trend for less blood product transfusion support during engraftment period. GVHD occurrence may be decreased and less severe with cotransplantation, translating to better morbidity and survival advantage. The risk of disease relapse and infection has been more controversial in the cotransplant setting with one report of increased risks in both that reached statistical significance [40] and a more recently published negative trial [44]. Both the clinical efficacy and potential risks will need to be confirmed in larger sample phase III studies. 

Perhaps even more relevant in the cotransplant setting is the understanding of potential interactions between MSC and immunosuppressive medications. Preclinical studies suggest detrimental effect of tacrolimus and rapamycin on MSC functions [45, 46]. However in an animal model of heart transplant, MSC combined with mycophenolate or rapamycin appears to have synergistic immunosuppressive effect [16]. More preclinical data is needed to inform better design of combined pharmacotherapy and cell therapy approach.

Given the consideration for risk of GVHD and cost and logistics of MSC cotransplant, it appears worth further exploration to determine whether high-risk patients receiving mismatched transplant and those with a history of graft rejection/failure may benefit from cotransplantation. Similar challenges exist with MSC therapy in cotransplant as with treatment of GVHD: no donor MSC engraftment was seen and mechanism of action is not clear.

## 4. Conclusions

In conclusion, clinical evidence suggests that MSC may have some activity in the treatment and prevention of GVHD; however the largest randomized trials have not confirmed this to date. A better understanding of the underlying biology is needed to rationally design further phase III trials attempting to confirm efficacy and clarify risk of disease relapse and infection. Optimization of MSC therapy in GVHD and other clinical conditions will require better understanding of these cells' mechanism of action and how these functions are affected by other existing treatment modalities. This knowledge can then be translated to improve the design of MSC therapy. Advances in personalized medicine should be employed to identify the patient population likely to benefit from MSC therapy and the role of MSC in combination with existing treatment modalities.

## Figures and Tables

**Table 1:** MSC for treatment of adult GVHD.

Ref	Phase	No. of pts	GVHD type	GVHD location	MSC source (no of doses)	MSC dose (×10^6^ cells/kg, median (range))	No of doses/pt (no of pt)	Time from Tplt (days, median (range))	Time from GVHD sx/dx (days, median (range))	Clinical response	TRM	Relapsed dz	Morbidity	Survival (after transplant)
[25]	Pilot/I	7	Acute (6): III (5), IV (1). Chronic (1): extensive.	S (1). G (1). S/G (3).	BM: MF (2), haplo (3), MM (3)	1.2 (0.6–2)	1 (6). 2 (1).	136.5 (32–283)	23 (7–90)	aGVHD (6): CR (5), NR (1). cGVHD (1): NR.	NA	1 from aGVHD group	Infection (2)	aGVHD: Alive (5), 2–36 mo; dead (2): 9 mo. cGVHD: dead (1), 12 mo.

[27]*	II	30	Acute (55): II (5), III (25), IV (25).*	S (3), H (1), G (6). S/G (15), H/G (7), S/H (4). S/H/G (19).*	BM: MF (5), haplo (18), MM (69).*	1.4 (0.4–9)*	1 (27), 2 (22), 3 (4), 4 (1), 5 (1).*	103 (27–533)*	NA	aGVHD (30): CR (13), PR (5), SD (1), PD (11).	1-yr from MSC: CR 37% (95% CI: 19–55%), non-CR 72% (95% CI: 55–89%), *P* = 0.002.*	3 from aGVHD group*	EBV PTLD (1).* development of extensive cGVHD (2).	Est. 2-yr survival: 26% (95% CI: 10–42%)

[28]	Pilot/I	3	Acute (3): III (3).	G (1), S/G (1), S/H/G (1)	BM: haplo (3)	0.51 (0.32–2)	1 (3)	120 (96–158)	92 (76–136)	aGVHD (3): NR (3).	NA	None	Infection (1).	aGVHD: dead (3), 177, 178, 199 days.

[29]	Pilot/I	4	Chronic (4): extensive.	Sclerodermatous (4).	BM: MM (23).	1.8 (1.0–2.0) × 10^7^ total cells/dose	4 (2), 7 (1), 8 (1).	13.8 (4.5–37.3) months	NA	cGVHD (4): PR (4).	NA	None	NA	cGVHD: alive (4), 4.6–23 mo.

[30]	I/II	18	Acute (10): II (3), III (2), IV (5). Chronic (8): limited (1), extensive (7)	G (8), S/G (5), S/M (1), G/M (1). S/H/G (3),	BM: MF (4), haplo (14).	1.0 (0.2–2.9)	1 (6), 2 (7), 3 (1), 4 (4).	NA	NA	aGVHD (10): CR(1) PR(6) NR(3) cGVHD (8): CR(1) PR(3) NR(4)	NA	2 from aGVHD group.	aGVHD group: infection (5), VOD (1).	aGVHD: alive (1), dead (9). cGVHD: alive (5), dead (3).

[31]	Pilot/I	13	Acute (13): III (2), IV (11)	G (2), S/G (1), S/H (2), H/G (5), S/H/G (3).	BM: MM (all).	0.9 (0.6–1.1)	1(1). 2(8) 3(2). 4(1) 5(1).	41 (20–91)	16 (4–31)	aGVHD (13): CR (1), PR (1), MR (5), NR (6).	NA	NA	Infection (3).	aGVHD: alive (4), 185–692 days; dead (9).

[32]	Pilot/I	6	Acute (6): III (2), IV (4).	H (1), S/H (2), S/G (2), S/H/G (1).	Adipose: haplo (2), MM (4).	1.0	1	71 (65–243)	38.5 (7–58)	aGVHD (6): CR (5), NR (1).	NA	1 from aGVHD group	No major infections reported.	aGVHD: alive (4), 18–90 mo; dead (2), 0.5, 13 mo.

[33]	II	31	De novo acute (31): II (21), III (7), IV (3).	S (13), G (11), multiorgan (7).	BM: MM (all Prochymal)	15 pts received dose of 2.0. 16 pts received dose of 8.0.	2	NA	within 48 hrs of diagnosis	aGVHD (31): CR (24), PR (5).	NA	aGVHD (3)	Infection (18)	aGVHD at 90 days: alive (22), dead (9).

Notes: *data from entire study including pediatric and adults.

GVHD locations: S: skin, H: hepatic, L: lung, G: gut. M: musculoskeletal.

Type of MSC: BM: bone marrow. Haplo: haploidentical donor. MF: HLA-identical family member. MUD: matched unrelated donor. MM: HLA-mismatched donor.

Clinical response: CR: complete response, PR: partial response, MR: minimal response, NR: no response. SD: stable disease. PD: progressive disease.

**Table 2:** MSC for treatment of pediatric GVHD.

Ref	Phase	No. of pts	GVHD type	GVHD location	Type of MSC (no of doses)	MSC dose (×10^6^ cells/kg, median (range))	No. of doses/pt (no of pts)	Time from Tplt (days, median (range))	Time from GVHD sx/dx (days, median (range))	Clinical response	TRM	Relapsed/ progressive primary disease	Morbidity	Survival (after transplant)
[25]	Pilot	2	acute (2): III (1), IV (1)	G (1). H/G (1).	BM: haplo (3), MM (1)	1.2 (0.7–2)	2 (2)	88.5 (73–170)	52, 63	aGVHD (2): CR (1), NR (1)	NA	NA	aGVHD: EBV PTLD (1)	aGVHD: dead (2), 3 mo, 12 mo.

[26]	Pilot/I	5	acute (2): II (1), III (1). Chronic (3): extensive (3).	S/G (1), S/H/G (3), S/H/L/G (1).	BM: MF (1), MUD (2), haplo (2).	2.0 (0.4–3.0)	1 (3), 2 (2).	250 (80–768)	NA	aGVHD (2): CR (1), NR (1). cGVHD (3): MR (1), NR (2).	NA	1 from aGVHD group.	cGVHD: EBV PTLD (1)	aGVHD: alive (1), 4 yrs; dead (1), NA. cGVHD: alive (1), 4 yrs; dead (2), 2.25–3 yrs,

[27]*	II	25	acute (55): II (5), III (25), IV (25).*	S (3), H (1), G (6). S/G (15, H/G (7), S/H (4). S/H/G (19).*	BM: MF (5), haplo (18), MM (69).*	1.4 (0.4–9)*	1 (27), 2 (22), 3 (4), 4 (1), 5 (1).*	103 (27–533)*	NA	aGVHD (25): CR (17), PR (4), SD (2), PD (2).	1-yr from MSC: CR 37% (95% CI: 19–55%), non-CR 72% (95% CI: 55–89%), *P* = 0.002.*	3 from aGVHD group*	EBV PTLD (1).*development of cGVHD: limited (2), extensive (4). De novo AML (1).	Est. 2-yr survival: 45% (95% CI: 23–67%)

[34]	Pilot/I	12	acute (12): III (5), IV (7).	G (5), S/G (3), H/G (2), S/H/G (1).	BM: MM (Prochymal)	Two pts: 8.0. Ten pts: 2.0.	2 (1), 3 (1), 7 (1), 8 (3), 9 (1), 12 (4), 21 (1).	98 (45–237)	46 (18–157)	aGVHD (12): CR (7), PR (2).	NA	NA	Infection (7), EBV PTLD (1).	Est. 2-yr survival: 40% (95% CI: 20–82%).

Notes: *data from entire study including pediatric and adults.

GVHD locations: S: skin, H: hepatic, L: lung, G: gut. M: musculoskeletal.

Type of MSC: BM: bone marrow. Haplo: haploidentical donor. MF: HLA-identical family member. MUD: matched unrelated donor. MM: HLA-mismatched donor.

Clinical response: CR: complete response, PR: partial response, MR: minimal response, NR: no response. SD: stable disease. PD: progressive disease.

**Table 3:** MSC cotransplantation clinical trials.

Ref	Phase	No. of pts	Type of Tplt (no of pts)	Conditioning Regimen (no. of pts)	CD34 dose. Other cells if specified (×10^6^ cells/kg, median (range)).	Other GVHD PPx	Type of MSC (no. of doses)	MSC dose (×10^6^ cells/kg, median (range))	Time to Tplt	Failed engraftment	Engraftment (days, median (range))	aGVHD (no/total evaluable (percent))	cGVHD (no/total evaluable (percent))	Relapsed disease (no/total (percent))	Infections (no/total (percent))	Morbidity/ mortality (percent (95% CI))	Survival (percent (95% CI))
[38]	I	46 (adults)	PBSCT, MF.	Bu/Cy, Thiopeta/Cy, BEAM, Cy/TBI	5.0 (3.2–15.8)	CSA & MTX (day 1, 3, 6).	MF, same as SCT donor.	2.5 (1–5)	4 hrs before SCT	0	ANC: 14 (11–26). Plt: 20.5 (15–36).	23/46 (50%). *⩾* grade II: 13/46 (28.3%)	22/36 (61%) Extensive: 8/36 (22.2%)	12/46 (26%)	NA	Est. 2-yr PFS: 53% (37–66%)	Est. 2-yr: 78% (63–87%)

[39]	I/II	14 (peds)	PBSCT, haplo.	TBI-based (9), chemo-based (5).	21.5 (11.6–38.6); CD3: 0.3 ± 0.3 × 10^5^ cells/kg.	NA	Haplo, same as SCT donor.	1.6 (1–3.3)	4 hrs before SCT	0	ANC: 12 (10–17). Plt: 10 (9–18).	2/14 (14.3%). Grade III-IV: 0.	1/7 (14.3%). Extensive: 0.	2/14 (14.3%)	Viral reacti-vation: 7/14 (50%). Fatal: 2/14 (14.3%)	Relapse-free survival at median 9 mo FU: 82%	Est. 9 mo: 72%

[40]	Pilot	10 (9 adults, 1 ped)	PBSCT (4), BM (4), PBSC & BM (2). All MF.	Cy/TBI (9), Bu/Cy (1).	5.14 (0.42–7.14)	CSA & MTX (day 1, 3, 6).	MF, same as SCT donor.	0.34 (0.03–1.53)	4 hrs before SCT	1	ANC: 16 (12–21). Plt > 50 × 10^9^/L: 30 (16–45).	4/9 (44.5%). Grade III-IV: 0.	1/7 (14.3%). Extensive: 0.	6/10 (60%)	3/10 (30%)	Est. 3-yr PFS: 30%	Est. 3-yr: 40%

[41]	Pilot/I	12 (adults)	PBSCT, MF.	Cy (2), Cy/TBI (8), Bu/Cy (2).	4.34 (2.59–13.1)	CSA & MTX (day 1, 3, 6, 11).	MF, same as SCT donor.	1.48 (1.29–2.24)	1 hr after SCT	0	ANC: 11 (7–15). Plt: 13.5 (10–17).	9/12 (75%). *⩾* grade II: 2/12 (16.7%).	4/12 (33.3%). Extensive: 2/12 (16.7%).	4/12 (33.3%)	Viral reacti-vation: 4/12 (30%). Fatal infection 1/12 (8.3%).	NA	Alive: 7/12, 58.3%, range of FU 29 to >57 mo. Dead: 5/12, 41.7%, range 5–19 mo post-tplt.

[42]	Pilot	7 (3 adults, 4 peds)	PBSCT (4): MF (2), MUD (2). BM (2): MF (1), MM (1). CB, MM (1).	Cy/TBI (1), Flu/ATG (2), Bu/Cy/ ATG (2), Flu/Mel/ ATG (1), Cy/TBI/ ATG/Cam (1).	7.2 (0.21–68.9)	CSA (2), CSA & MTX (3), CSA & Pred (1), Tac & Rapamycin (1).	MF, same as SCT donor (3). Haplo (4).	1.0	Within 4 hrs of SCT	0	ANC: 12 (10–28). Plt > 50 × 10^9^/L: 15 (11–45).	6/7 (85.7%). Grade II: 2/7 (28.6%). Grade III-IV: 0.	1/7 (14.3%), extensive.	0/7 (0%).	Severe infections: 2/7 (28.3%). Fatal: 1/7 (14.3%).	PFS at 2-yr median FU: 6/6 (100%).	2-yr median FU: 6/7 (85.7%).

[43]	I/II	9 (adults)	CB & PBSCT: PBSCT haplo (7), MM (2).	Cy/TBI/ATG /Flu (6), Bu/Cy /Flu/ATG (3).	PBSCT: 2.61 (2.4–3.31). CB: 0.12 (0.037–0.28). CD3: 0.0036 (0.0001–0.0083).	CSA & Methyl-pred (day-1 to 10–14).	same as SCT donors.	1.2 (1.04–2.22)	CB, then PB-SCT, then MSC within 24 hrs.	0	ANC: 12 (10–31). Plt: 44 (27–98).	5/9 (55.6%). Grade II: 4/9 (44.4%). Grade III-IV: 0.	1/8 (12.5%), limited.	1/9 (11.1%)	Viral reacti-vation: 2/9 (22.2%).	2-yr cumulative TRM: 11% (2–71%)	OS at 22 mo: 8/9 (88.9%).

[44]	Pilot/I	20 (adults)	PBSCT, MM.	Flu/TBI	4.8 (1.6–11.8). CD3: 312 (120–540).	MMF & Tac	MM, third party different from SCT donors.	NA	0.5–2 hrs before SCT	1	NA	11/20 (55%). Grade II: 5/20 (25%). Grade III-IV: 4/20 (20%).	15/20 (75%). Extensive: 8/20 (40%).	6/20 (30%)	Fatal: 1/20 (5%).	1-yr PFS: 60%.	1-yr OS: 80%.

Notes: Type of transplant (tplt). BM: bone marrow stem cells. CB: cord blood stem cells. Haplo: haploidentical donor. MF: HLA-identical family member. MUD: matched unrelated donor. MM: HLA-mismatched donor. PBSCT: peripheral blood stem cells.

Conditioning regimen: ATG: antithymoglobulin. BEAM: carmustine, etoposide, cytarabine, melphalan. Bu: busulfan. Cam: campath. Cy: cyclophosphamide. Flu: fludarabine. Mel: melphalan. TBI: total body irradiation. CSA: cyclosporin. MMF: mycophenolate mofetil. MTX: methotrexate. Tac: tacrolimus.

GVHD prophylaxis (PPX): CSA: cyclosporin. MMF: mycophenolate mofetil. MTX: methotrexate. Tac: tacrolimus.

Engraftment criteria unless otherwise specified: time needed to reach ANC > 0.5 × 10^9^/L. Plt > 20 × 10^9^/L.

## References

[1] Horwitz EM, Le Blanc K, Dominici M (2005). Clarification of the nomenclature for MSC: the international society for cellular therapy position statement. *Cytotherapy*.

[2] Dominici M, Le Blanc K, Mueller I (2006). Minimal criteria for defining multipotent mesenchymal stromal cells. The international society for cellular therapy position statement. *Cytotherapy*.

[3] Selmani Z, Naji A, Zidi I (2008). Human leukocyte antigen-G5 secretion by human mesenchymal stem cells is required to suppress T lymphocyte and natural killer function and to induce CD4^+^ CD25^high^FOXP3^+^ regulatory T cells. *Stem Cells*.

[4] Bartholomew A, Sturgeon C, Siatskas M (2002). Mesenchymal stem cells suppress lymphocyte proliferation in vitro and prolong skin graft survival in vivo. *Experimental Hematology*.

[5] Di Nicola M, Carlo-Stella C, Magni M (2002). Human bone marrow stromal cells suppress T-lymphocyte proliferation induced by cellular or nonspecific mitogenic stimuli. *Blood*.

[6] Le Blanc K, Tammik L, Sundberg B, Haynesworth SE, Ringden O (2003). Mesenchymal stem cells inhibit and stimulate mixed lymphocyte cultures and mitogenic responses independently of the major histocompatibility complex. *Scandinavian Journal of Immunology*.

[7] Tse WT, Pendleton JD, Beyer WM, Egalka MC, Guinan EC (2003). Suppression of allogeneic T-cell proliferation by human marrow stromal cells: implications in transplantation. *Transplantation*.

[8] Aggarwal S, Pittenger MF (2005). Human mesenchymal stem cells modulate allogeneic immune cell responses. *Blood*.

[9] Meisel R, Zibert A, Laryea M, Gobel U, Däubener W, Dilloo D (2004). Human bone marrow stromal cells inhibit allogeneic T-cell responses by indoleamine 2, 3-dioxygenase-mediated tryptophan degradation. *Blood*.

[10] Jiang XX, Zhang Y, Liu B (2005). Human mesenchymal stem cells inhibit differentiation and function of monocyte-derived dendritic cells. *Blood*.

[11] Corcione A, Benvenuto F, Ferretti E (2006). Human mesenchymal stem cells modulate B-cell functions. *Blood*.

[12] Spaggiari GM, Capobianco A, Becchetti S, Mingari MC, Moretta L (2006). Mesenchymal stem cell-natural killer cell interactions: evidence that activated NK cells are capable of killing MSCs, whereas MSCs can inhibit IL-2-induced NK-cell proliferation. *Blood*.

[13] Kinnaird T, Stabile E, Burnett MS (2004). Marrow-derived stromal cells express genes encoding a broad spectrum of arteriogenic cytokines and promote In Vitro and In Vivo arteriogenesis through paracrine mechanisms. *Circulation Research*.

[14] Haynesworth SE, Baber MA, Caplan AI (1996). Cytokine expression by human marrow-derived mesenchymal progenitor cells in vitro: effects of dexamethasone and IL-1*α*. *Journal of Cellular Physiology*.

[15] Majumdar MK, Thiede MA, Haynesworth SE, Bruder SP, Gerson SL (2000). Human marrow-derived mesenchymal stem cells (MSCs) express hematopoietic cytokines and support long-term hematopoiesis when differentiated toward stromal and osteogenic lineages. *Journal of Hematotherapy and Stem Cell Research*.

[16] Popp FC, Eggenhofer E, Renner P (2008). Mesenchymal stem cells can induce long-term acceptance of solid organ allografts in synergy with low-dose mycophenolate. *Transplant Immunology*.

[17] Ge W, Jiang J, Baroja ML (2009). Infusion of mesenchymal stem cells and rapamycin synergize to attenuate alloimmune responses and promote cardiac allograft tolerance. *American Journal of Transplantation*.

[18] Casiraghi F, Azzollini N, Cassis P (2008). Pretransplant infusion of mesenchymal stem cells prolongs the survival of a semiallogeneic heart transplant through the generation of regulatory T cells. *Journal of Immunology*.

[19] Yanez R, Lamana ML, Garcia-Castro J, Colmenero I, Ramirez M, Bueren JA (2006). Adipose tissue-derived mesenchymal stem cells have in vivo immunosuppressive properties applicable for the control of the graft-versus-host disease. *Stem Cells*.

[20] Sudres M, Norol F, Trenado A (2006). Bone marrow mesenchymal stem cells suppress lymphocyte proliferation in vitro but fail to prevent graft-versus-host disease in mice. *Journal of Immunology*.

[21] Polchert D, Sobinsky J, Douglas GW (2008). IFN-*γ* activation of mesenchymal stem cells for treatment and prevention of graft versus host disease. *European Journal of Immunology*.

[22] Ren G, Su J, Zhang L (2009). Species variation in the mechanisms of mesenchymal stem cell-mediated immunosuppression. *Stem Cells*.

[23] Ferrara JL, Levine JE, Reddy P, Holler E (2009). Graft-versus-host disease. *The Lancet*.

[24] Le Blanc K, Rasmusson I, Sundberg B (2004). Treatment of severe acute graft-versus-host disease with third party haploidentical mesenchymal stem cells. *The Lancet*.

[25] Ringden O, Uzunel M, Rasmusson I (2006). Mesenchymal stem cells for treatment of therapy-resistant graft-versus-host disease. *Transplantation*.

[26] Muller I, Kordowich S, Holzwarth C (2008). Application of multipotent mesenchymal stromal cells in pediatric patients following allogeneic stem cell transplantation. *Blood Cells, Molecules, and Diseases*.

[27] Le Blanc K, Frassoni F, Ball L (2008). Mesenchymal stem cells for treatment of steroid-resistant, severe, acute graft-versus-host disease: a phase II study. *The Lancet*.

[28] Arima N, Nakamura F, Fukunaga A (2010). Single intra-arterial injection of mesenchymal stromal cells for treatment of steroid-refractory acute graft-versus-host disease: a pilot study. *Cytotherapy*.

[29] Zhou H, Guo M, Bian C (2010). Efficacy of bone marrow-derived mesenchymal stem cells in the treatment of sclerodermatous chronic graft-versus-host disease: clinical report. *Biology of Blood and Marrow Transplantation*.

[30] Perez-Simon JA, Lopez-Villar O, Andreu EJ (2011). Mesenchymal stem cells expanded in vitro with human serum for the treatment of acute and chronic graft-versus-host disease: results of a phase I/II clinical trial. *Haematologica*.

[31] von Bonin M, Stolzel F, Goedecke A (2009). Treatment of refractory acute GVHD with third-party MSC expanded in platelet lysate-containing medium. *Bone Marrow Transplantation*.

[32] Fang B, Song Y, Liao L, Zhang Y, Zhao RC (2007). Favorable response to human adipose tissue-derived mesenchymal stem cells in steroid-refractory acute graft-versus-host disease. *Transplantation Proceedings*.

[33] Kebriaei P, Isola L, Bahceci E (2009). Adult human mesenchymal stem cells added to corticosteroid therapy for the treatment of acute graft-versus-host disease. *Biology of Blood and Marrow Transplantation*.

[34] Prasad VK, Lucas KG, Kleiner GI (2010). Efficacy and safety of ex vivo cultured adult human mesenchymal stem cells (Prochymal) in pediatric patients with severe refractory acute graft-versus-host disease in a compassionate use study. *Biology of Blood and Marrow Transplantation*.

[35] Reagan MR, Kaplan DL (2011). Concise review: mesenchymal stem cell tumor-homing: detection methods in disease model systems. *Stem Cells*.

[36] Parekkadan B, Milwid JM (2010). Mesenchymal stem cells as therapeutics. *Annual Review of Biomedical Engineering*.

[37] Lee ST, Jang JH, Cheong JW (2002). Treatment of high-risk acute myelogenous leukaemia by myeloablative chemoradiotherapy followed by co-infusion of T cell-depleted haematopoietic stem cells and culture-expanded marrow mesenchymal stem cells from a related donor with one fully mismatched human leucocyte antigen haplotype. *British Journal of Haematology*.

[38] Lazarus HM, Koc ON, Devine SM (2005). Cotransplantation of HLA-identical sibling culture-expanded mesenchymal stem cells and hematopoietic stem cells in hematologic malignancy patients. *Biology of Blood and Marrow Transplantation*.

[39] Ball LM, Bernardo ME, Roelofs H (2007). Cotransplantation of ex vivo-expanded mesenchymal stem cells accelerates lymphocyte recovery and may reduce the risk of graft failure in haploidentical hematopoietic stem-cell transplantation. *Blood*.

[40] Ning H, Yang F, Jiang M (2008). The correlation between cotransplantation of mesenchymal stem cells and higher recurrence rate in hematologic malignancy patients: outcome of a pilot clinical study. *Leukemia*.

[41] Zhang X, Li JY, Cao K (2010). Cotransplantation of HLA-identical mesenchymal stem cells and hematopoietic stem cells in Chinese patients with hematologic diseases. *International Journal of Laboratory Hematology*.

[42] Le Blanc K, Samuelsson H, Gustafsson B (2007). Transplantation of mesenchymal stem cells to enhance engraftment of hematopoietic stem cells. *Leukemia*.

[43] Gonzalo-Daganzo R, Regidor C, Martin-Donaire T (2009). Results of a pilot study on the use of third-party donor mesenchymal stromal cells in cord blood transplantation in adults. *Cytotherapy*.

[44] Baron F, Lechanteur C, Willems E (2010). Cotransplantation of mesenchymal stem cells might prevent death from graft-versus-host disease (GVHD) without abrogating graft-versus-tumor effects after HLA-mismatched allogeneic transplantation following nonmyeloablative conditioning. *Biology of Blood and Marrow Transplantation*.

[45] Hoogduijn MJ, Crop MJ, Korevaar SS (2008). Susceptibility of human mesenchymal stem cells to tacrolimus, mycophenolic acid, and rapamycin. *Transplantation*.

[46] Buron F, Perrin H, Malcus C (2009). Human mesenchymal stem cells and immunosuppressive drug interactions in allogeneic responses: an In Vitro study using human cells. *Transplantation Proceedings*.

